# Persistence, adherence, healthcare resource utilisation and costs for interferon Beta in multiple sclerosis: a population-based study in the Campania region (southern Italy)

**DOI:** 10.1186/s12913-020-05664-x

**Published:** 2020-08-26

**Authors:** Marcello Moccia, Ilaria Loperto, Roberta Lanzillo, Antonio Capacchione, Antonio Carotenuto, Maria Triassi, Vincenzo Brescia Morra, Raffaele Palladino

**Affiliations:** 1 Multiple Sclerosis Clinical Care and Research Centre, Department of Neuroscience, Reproductive Science and Odontostomatology, Federico II University, Via Sergio Pansini 5, Building 17, Ground floor, 80131 Naples, Italy; 2 Department of Public Health, Federico II University, Naples, Italy; 3 Merck Serono S.p.A (an affiliate of Merck KGaA, Darmstadt, Germany), Rome, Italy; 4 Department of Primary Care and Public Health, Imperial College, London, UK

**Keywords:** Multiple sclerosis, Interferon, Healthcare resource utilization, Costs

## Abstract

**Background:**

To differentiate five formulations of Interferon Beta for the treatment of multiple sclerosis (MS) in clinical practice, by analysing persistence, adherence, healthcare resource utilisation and costs at population level.

**Methods:**

In this population-based study, we included individuals with MS living in the Campania Region of Italy from 2015 to 2017, on treatment with intramuscular Interferon Beta-1a (Avonex® = 618), subcutaneous pegylated Interferon Beta-1a (Plegridy® = 259), subcutaneous Interferon Beta-1a (Rebif® = 1220), and subcutaneous Interferon Beta-1b (Betaferon® = 348; and Extavia® = 69). We recorded healthcare resource utilisation from administrative databases (hospital discharges, drug prescriptions, MS-related outpatients), and derived costs from the Regional formulary. We classified hospital admissions into MS-related and non-MS-related. Persistence (time to switch to other disease modifying treatments (DMTs)), and adherence (medication possession ratio (MPR) = medication supply obtained/medication supply expected during follow-up period) were calculated.

**Results:**

Patients treated with Rebif® were younger, when compared with other Interferon Beta formulations (*p* < 0.01). The probability of switching to other DMTs was 60% higher for Betaferon®, 90% higher for Extavia®, and 110% higher for Plegridy®, when compared with Rebif® (*p* < 0.01). Plegridy® presented with 7% higher adherence (*p* < 0.01), and Betaferon® with 3% lower adherence (*p* = 0.03), when compared with Rebif®. The probability of MS-related hospital admissions was 40% higher in Avonex® (*p* = 0.03), 400% higher in Betaferon® (*p* < 0.01), and 60% higher in Plegridy® (*p* = 0.04), resulting into higher non-DMT-related costs, when compared with Rebif®.

**Discussion:**

Interferon Beta formulations presented with different prescription patterns, persistence, adherence, healthcare resource utilisation and costs, with Rebif® being used in younger patients and with less MS-related hospital admissions.

## Background

Multiple sclerosis (MS) is an immune-mediated disease of the central nervous system, and represents the most common cause of neurological disability among young adults [1, 2].

In the past decades, fifteen injectable, oral, and monoclonal antibody disease-modifying therapies (DMTs) have become available for MS [3], with significant financial burden on the healthcare systems [4]. Among DMTs, there are five preparations of Interferon Beta, which are different in terms of dosing and administration (e.g., frequency, subcutaneous/intramuscular injection, associated devices), but are difficult to characterise in clinical practice, with few studies performing direct comparison [1, 3]. A number of meta-analyses of randomised controlled trials (RCTs) have shown that the use of some Interferon Beta-1a formulations can mitigate the risk of disability progression, despite no differences in relapse risk [5–8]. However, there are limitations to be accounted for when considering RCTs, characterised by a short follow-up (24 months), highly selected populations, and the use of placebo as comparator group. Real-world studies also suggested Interferon Beta formulations can exert long-term beneficial effects on disability outcomes [9, 10]. Still, in clinical registries, there are potential risks from patient selection (e.g., inclusion of patients and clinical variables only from participating centres), and follow-up (e.g., variable follow-up duration, with patients doing poorly being most likely to be lost to follow-up) [11, 12]. Not least, both RCTs and clinical registries do not include healthcare resource utilisation and, more in general, the complexity of MS management [3, 13, 14].

In the present population-based study, we used routinely collected healthcare data in the Campania Region of Italy, from 2015 to 2017, to describe the use of different Interferon Beta formulations and to evaluate possible differences, assuming that dosing, frequency of administration, subcutaneous/intramuscular injection, and/or associated devices can affect persistence, adherence, healthcare resource utilisation and costs.

## Methods

### Study design and setting

In this population-based study, we performed a retrospective analysis on routinely collected healthcare data, prospectively recorded from 2015 to 2017, on individuals with a diagnosis of MS living in the Campania Region of Italy (representing 10% of the Italian population). The original dataset has been described elsewhere [15].

DMT prescriptions in the Campania Region are provided to individuals with MS by ten qualified MS centres, complying with regulatory indications for DMT prescription and management [16–18], and are refunded by the Italian National Healthcare Service (NHS). Healthcare services delivered out of the Campania Region (e.g., DMT prescription, inpatient, outpatient) are then reported to the Campania Region for refund purposes. As such, healthcare resource utilisation for individuals with MS living in the Campania Region, is entirely traceable by the Campania Region Healthcare Regulatory Society (So.Re.Sa.).

The study was approved by the Federico II Ethics Committee (355/19). All patients signed informed consent authorising the use of anonymised data collected routinely as part of the clinical practice, in line with data protection regulation (GDPR EU2016/679). The study was performed in accordance with good clinical practice and Declaration of Helsinki.

### Population

Dataset was created by merging different data sources of the Campania Region, as fully described elsewhere [15]. In particular, the cohort comprised all individuals resident in the Campania Region who had at least one MS record, from 2015 to 2017, in the Hospital Discharge Record database, the Regional Drug Prescription database, or the outpatient database with payment exemptions for MS. The case-finding algorithm identified 5362 MS cases, with 99.0% sensitivity [15].

For the purposes of the present study, we included individuals with a diagnosis of MS receiving at least one Interferon Beta prescription during the study period, covering at least 3 months, and, for statistical purposes, we specifically referred to individual treatment periods, since the same patient could have been using different Interferon Beta formulations during the study period. Interferon Beta formulations included in the present study were: intramuscular Interferon Beta-1a (Avonex®), subcutaneous pegylated Interferon Beta-1a (Plegridy®), subcutaneous Interferon Beta-1a (Rebif®; available in both 22 μg and 44 μg formulations), and subcutaneous Interferon Beta-1b (Betaferon® and Extavia®).

From the database, individuals with a diagnosis of MS not resident in the Campania Region of Italy were filtered out. Patient unique identifier code was fully anonymised by the Campania Region regulatory agency before releasing the datasets. As the same anonymisation algorithm was used across datasets, data merging was possible, and the anonymised patient id attributed to a patient in one dataset was the same attributed in another dataset. As additional measure of privacy protection, the only demographic information retained from each dataset were: year of birth, sex, education attainment, and local health authority the individual is registered with.

For patients with Hospital Discharge Records, Charlson Comorbidity Index was computed, as in previous population-based studies in MS [19]. The Charlson Comorbidity Index assigns different weights to comorbidities reported with ICD codes in Hospital Discharge Records. The overall score is obtained from the sum of different weights, and provides the risk of death from comorbidities [20].

### Persistence and adherence

Persistence was measured as the time spent on a specific DMT (related to each individual treatment period). In accordance with previous studies on the same topic, discontinuation of Interferon Beta treatment was defined as a > 90-day interruption in therapy, a switch to another DMT (i.e., Glatiramer acetate, Natalizumab, or Fingolimod), or complete discontinuation (i.e., no further record of medication initiation) [21–24]. When considering different Interferon Beta treatments, switching from one Interferon Beta-1a dose to another (e.g., from Avonex® to Rebif®), or from Interferon Beta-1a to Beta-1b formulations was considered as discontinuation [21–24]. We specifically evaluated switch to another DMT, whilst did not consider patients with interruption of DMTs and/or without further record of DMT initiation, which could have occurred for a number of reasons our dataset is currently not able to account for (e.g., transfer to another Region, pregnancy, death).

Medication possession ratio (MPR) was calculated as an indirect measure of adherence (MPR = (medication supply obtained during follow-up period/medication supply expected during follow-up period)*100) [25], and was referred to individual treatment periods. Medication supply was electronically checked in the clinical practice, thus limiting the risk of overestimating adherence [25].

### Healthcare resource utilisation and costs

Healthcare resource utilisation was obtained from the combination, for each individual treatment period, of hospital discharge records (including all hospital admissions and diagnoses), drug prescriptions (including the frequency of Interferon Beta prescriptions), and MS-related outpatient consultations. We further classified hospital admissions into regular inpatients and emergency admissions, and into MS-related and non-MS-related admissions, based on the main discharge diagnosis [26].

Healthcare costs were derived from the Regional registry for corresponding healthcare resource utilisation [27]. Healthcare costs were inflated to the most recent values (2017), in order to avoid variations in price per unit of service through different years, and were reported on a monthly basis (total costs during the individual treatment period / months of follow-up).

#### Statistics

Different Interferon Beta formulations were considered as the main variable of interest (Rebif®, Avonex®, Plegridy®, Betaferon®, Extavia®). Distribution of variables was assessed, and descriptive statistics were performed using chi-square test (i.e., sex distribution), or analysis of variance (ANOVA) (i.e., age, Charlson comorbidity index).

Differences in persistence between Interferon Beta formulations (individual treatment periods) were evaluated using Cox regression models (automatically accounting for the time of observation of each individual). Differences in adherence and costs between Interferon Beta formulations (individual treatment periods) were evaluated using mixed-effect linear regression models. Differences in healthcare resource utilisation between Interferon Beta formulations (individual treatment periods) were evaluated using mixed-effect logistic regression models. Considering that across the study period a proportion of individuals changed of Interferon Beta formulation, we specifically used mixed-effect models where each individual was included as random intercept.

Covariates were age, sex, and treatment duration (time from first prescription to discontinuation or final follow-up date); the latter covariate was specifically selected to reduce the impact of different follow-up duration on study endpoints. Rebif® was considered as reference in the statistical models, since this represented the largest treatment subgroup.

Results are reported as hazard ratio (HR), coefficient (Coeff), odds ratio (OR), 95% confidence intervals (95%CI), and *p*-values, as appropriate. Statistical analyses were performed using Stata 15.0. Results were considered statistically significant for *p* < 0.05.

#### Data availability

Data is available upon request to Regional Healthcare Society (So.Re.Sa – www.soresa.it).

## Results

During the study period (2015–2017), among 5362 individuals with MS living in the Campania Region, we included 2171 individuals (40.4%) who received at least one Interferon Beta prescription, which resulted into 2514 individual treatment periods (open cohort). Distribution between Interferon Beta formulations is presented in Table 1, along with age and sex. Patients treated with Rebif® were younger, when compared with other Interferon Beta formulations (*p* < 0.01). No differences were detected for sex distribution (*p* = 0.81), and Charlson comorbidity index (*p* = 0.21).

**Table 1:** Utilisation of different Interferon Beta formulations

	**Avonex®**	**Betaferon®**	**Extavia®**	**Plegridy®**	**Rebif®**
**Number**	618	348	69	259	1220
**Age,** *years*	45.1 ± 13.8	48.1 ± 13.2	49.7 ± 14.2	42.1 ± 17.7	41.7 ± 16.0
**Sex,** *females (%)*	425 (68.77%)	211 (60.63%)	49 (71.01%)	178 (68.72%)	806 (66.06%)
**Charlson comorbidity index**					
*0*	301	172	43	107	632
*1–2*	27	32	8	6	35
*> 2*	3	3	1	3	7

Table shows the number of individual treatment periods for different Interferon Beta formulations, along with age, sex distribution (with percentage), and Charlson comorbidity index (for patients with hospital records). The cohort included 2171 individuals, overall resulting into 2514 individual treatments with Interferon Beta

### Persistence and adherence

From 2015 to 2017, 1115 patients (44.3%) discontinued their Interferon Beta formulation, after average time of 20.0 ± 11.8 months. Number of discontinuations of different Interferon Beta formulations are reported in Table 2, along with further DMT records (with time to switch and DMTs individuals were switched to). The probability of switching to other DMTs was 60% higher for Betaferon®, 90% higher for Extavia®, and 110% higher for Plegridy®, when compared with Rebif® (Table 2).

**Table 2:** Switching from Interferon Beta

	**Avonex®**	**Betaferon®**	**Extavia®**	**Plegridy®**	**Rebif®**
**Discontinuation**	257/618 (41.6%)	175/348 (50.2%)	38/69 (55.0%)	102/259 (39.3%)	543/1220 (44.4%)
**No further DMT record**	45/618 (7.3%)	45/348 (12.9%)	7/69 (10.1%)	23/259 (8.8%)	180/1220 (14.7%)
**Switch to other DMT**	212/618 (34.3%)	130/348 (37.3%)	31/69 (44.9%)	79/259 (30.5%)	363/1220 (29.7%)
**After***, months*	23.2 ± 12.7	22.5 ± 12.6	19.3 ± 13.2	11.7 ± 8.1	23.3 ± 12.6
**HR**	1.12	1.59	1.89	2.13	*reference*
**95%CI**	0.93, 1.34	1.30, 1.96	1.29, 2.77	1.59, 2.85	
***p*-value**	0.20	< 0.01	< 0.01	< 0.01	
**To:**	**(out of 212)**	**(out of 130)**	**(out of 31)**	**(out of 79)**	**(out of 363)**
**Aubagio**®	32 (15.1%)	26 (20.0%)	6 (19.3%)	6 (7.6%)	35 (9.6%)
**Avonex**®	–	4 (3.1%)	–	19 (24.0%)	9 (2.5%)
**Betaferon**®	2 (0.9%)	–	6 (19.3%)	3 (3.8%)	4 (1.1%)
**Copaxone**®	10 (4.7%)	7 (5.4%)	–	12 (15.2%)	12 (3.3%)
**Extavia**®	–	2 (1.5%)	–	–	–
**Gilenya**®	40 (18.9%)	26 (20.0%)	5 (16.1%)	10 (12.7%)	98 (27.0%)
**Lemtrada**®	–	–	–	–	1 (0.3%)
**Plegridy**®	72 (34.0%)	11 (8.5%)	3 (9.7%)	–	45 (12.4%)
**Rebif**®	7 (3.3%)	2 (1.5%)	3 (9.7%)	4 (5.1%)	–
**Tecfidera**®	44 (20.7%)	45 (34.6%)	7 (22.6%)	22 (27.8%)	131 (36.1%)
**Tysabri**®	5 (2.4%)	7 (5.4%)	1 (3.3%)	3 (3.8%)	27 (7.4%)

Table shows number and percentage of individual treatment periods with Interferon Beta discontinuation. Number and percentage of individual treatment periods is also reported for those without further DMT record, and for those switching to another DMT (with time to switch and DMTs individuals were switched to). HR, 95%CI, and *p*-values are reported from Cox regression models evaluating the rate of switch for different Interferon Beta formulations; covariates were age, sex, and treatment duration (Rebif® was used as reference in the statistical models)

Adherence rates (MPR) are reported in Table 3, and were, on average, above 80%. Among Interferon Beta formulations, Plegridy® presented with 7% higher adherence, and Betaferon® with 3% lower adherence, when compared with Rebif® (Table 3).

**Table 3:** Adherence (MPR) in different Interferon Beta formulations

	**Avonex®**	**Betaferon®**	**Extavia®**	**Plegridy®**	**Rebif®**
**MPR**	89.1 ± 2.5%	84.5 ± 2.9%	88.1 ± 2.6%	100. ± 3.4%	86.9 ± 3.0%
**Coeff**	0.83	−3.53	−1.07	7.43	*reference*
**95%CI**	−1.87, 3.53	−6.84, −0.23	−8.40, 6.24	2.79, 12.07	
***p*-value**	0.54	0.03	0.77	< 0.01	

Table shows adherence measured as MPR, in different Interferon Beta formulations. Coeff, 95%CI, and *p*-values are reported from mixed effect linear regression models adjusted by age, sex, and treatment duration (Rebif® was used as reference in the statistical models)

### Healthcare resource utilisation and costs

Out of all individuals with MS treated with different Interferon Beta formulations, 3.04% had inpatient admissions during treatment, and 2.03% had emergency admissions; 36.33% hospital admissions were MS-related. The probability of inpatient admissions was 300% higher in Extavia®, when compared with Rebif®; the probability of emergency admissions was 200% higher in Extavia® and Plegridy®, when compared with Rebif®; the probability of MS-related hospital admissions was 40% higher in Avonex®, 400% higher in Betaferon® and 60% higher in Plegridy®, when compared with Rebif® (Table 4).

**Table 4:** Differences in healthcare resource utilisation between different Interferon Beta formulations

	**Avonex®**	**Betaferon®**	**Extavia®**	**Plegridy®**	**Rebif®**
**Hospital admissions**	3.24%	2.86%	5.80%	2.77%	2.96
**OR**	0.97	1.03	3.80	0.72	*reference*
**95%CI**	0.41, 2.25	0.37, 2.85	1.27, 11.85	0.16, 3.11	
***p*-value**	0.95	0.94	0.02	0.66	
**Emergency admissions**	1.94%	2.3%	4.35%	2.54%	1.98%
**OR**	0.96	1.07	2.60	2.08	*reference*
**95%CI**	0.40, 2.27	0.43, 2.69	1.17, 4.17	1.08, 4.63	
***p*-value**	0.93	0.87	< 0.01	0.04	
**MS-related admissions**	38.51%	53.74%	31.88%	37.06%	34.90%
**OR**	1.41	4.77	1.22	1.67	*reference*
**95%CI**	1.07, 2.04	2.74, 8.31	0.48, 3.15	1.12, 3.01	
***p*-value**	0.03	< 0.01	0.67	0.04	

Table shows percent of patients with hospital admissions, emergency admissions, and MS-related admissions for different Interferon Beta formulations (out of all individuals on treatment with each Interferon Beta formulation). OR, 95%CI, and *p*-values are reported from mixed effect logistic regression models adjusted by age, sex, and treatment duration (Rebif® was used as reference in the statistical models)

On monthly basis, overall healthcare costs were lower for Avonex®, Betaferon®, Extavia®, and Plegridy®, when compared with Rebif®. Costs for DMTs were largely responsible for overall healthcare costs, and were lower for Avonex®, Betaferon®, and Extavia®, when compared with Plegridy® and Rebif®. Costs for MS-related outpatient consultations were higher for Avonex®, and costs for inpatient admissions were higher for Betaferon®, and lower for Plegridy®, when compared with Rebif®. Full results are reported in Table 5.

**Table 5:** Differences in total healthcare costs between different Interferon Beta formulations

**Monthly costs:**	**Avonex®**	**Betaferon®**	**Extavia®**	**Plegridy®**	**Rebif®**
**Overall costs (€)**	799.25 ± 287.98	465.75 ± 138.81	533.54 ± 339.93	824.75 ± 416.42	901.39 ± 441.48
**Coeff**	− 92.74	− 430.00	− 397.05	−99.00	*reference*
**95%CI**	137.38, −48.09	−467.04, − 392.97	− 474.24, − 319.87	− 175.96, −22.03	
***p*-value**	< 0.01	< 0.01	< 0.01	0.01	
**DMT costs (€)**	778.11 ± 283.90	448.57 ± 132.35	506.94 ± 329.90	822.88 ± 416.14	881.86 ± 431.02
**Coeff**	−94.99	− 419.33	− 411.00	−72.88	*reference*
**95%CI**	−137.38, −52.59	− 453.51, − 385.15	− 472.49, − 349.51	− 148.48, 2.71	
***p*-value**	< 0.01	< 0.01	< 0.01	0.06	
**Outpatient costs (€)**	0.95 ± 3.19	0.82 ± 1.61	0.79 ± 1.83	0.28 ± 1.67	0.69 ± 1.94
**Coeff**	0.46	−0.05	0.17	−0.68	*reference*
**95%CI**	0.00, 0.93	−0.35, 0.24	− 0.60, 0.96	−1.15, 0.22	
***p*-value**	0.04	0.72	0.65	0.83	
**Inpatient costs (€)**	20.19 ± 65.11	25.35 ± 44.31	25.80 ± 111.22	1.87 ± 0.94	18.84 ± 8.80
**Coeff**	1.78	10.62	13.77	−25.43	*reference*
**95%CI**	−10.87, 14.43	0.97, 22.21,	−35.72, 63.25	−37.28, − 13.59	
***p*-value**	0.78	0.04	0.58	< 0.01	

Table shows monthly costs for different Interferon Beta formulations, and, then, specific costs for DMTs, MS-related outpatients and inpatients. Coeff, 95%CI, and *p*-values are reported from mixed effect linear regression models adjusted by age, sex, and treatment duration (Rebif® was used as reference in the statistical models)

## Discussion

In the present population-based study, conducted from 2015 to 2017, we found that Interferon Beta was used in 40% MS patients of the Campania Region (Southern Italy). Rebif® was the most-commonly-used formulation, representing 48% of individual prescriptions. Overall, our study highlighted that Interferon Beta formulations have different prescription patterns, persistence, adherence, healthcare resource utilisation and costs.

The main strength of the present study is the comprehensive approach for studying MS treatments. Indeed, we included both DMTs and healthcare resource utilisation from routinely collected healthcare data, whilst previous studies mostly focused on DMTs, being the main responsible for direct medical costs, but providing a limited view on MS management [4, 28–30]. Our case-finding algorithm has 99.0% sensitivity, and is applied on 10% of the Italian population [15]. Of note, our demographics and Interferon Beta prescription rates are in line with international studies conducted within the same time frame, on equally sized populations [26, 31–34], thus suggesting overall generalisability of current findings.

Looking at demographics, Avonex®, Betaferon®, Extavia® and Plegridy® were preferred in older patients, whilst Rebif® was prescribed to younger patients (likely in the earlier phases of MS), where the cost-effectiveness of Interferon Beta is the highest, due to higher levels of disease activity [35]. This hypothesis is further supported by the switching pattern, showing that 15–25% MS patients on Avonex®, Betaferon® and Plegridy®, and 35% on Rebif® switched to DMTs with higher efficacy profile, suggesting the latter was used in more active patients, thus delaying the use of more expensive second-line DMTs [36, 37]. This prescription pattern could be due to the evidence suggesting higher efficacy of Rebif®, when compared with other Interferon Beta formulations [5, 10, 23].

Discontinuation of Interferon Beta treatment was observed in 30–45% patients over 20 months, with switch to other DMTs occurring in most cases. As such, persistence rates in our study were relatively higher, when compared with previous studies [38], suggesting overall good patient profiling [10]. Some previous studies failed to find any difference in persistence between Interferon Beta formulations [31], whilst others reported on discording results [23, 39]. Hereby, we found higher probability of switching to other DMTs for Betaferon®, Extavia® and Plegridy®, when compared with Rebif® and Avonex®. Reasons for Interferon Beta discontinuation are unfortunately not traceable in routinely-collected healthcare data, though possibly resulting from a combination of poor tolerance/side effects (accounting for up to 60% of discontinuation reasons), and lack of efficacy [23, 24], and could be only derived from the switching pattern indirectly. For instance, in our cohort, lower persistence to Plegridy® could be attributed to poor tolerance and/or higher number of side effects, since most patients switched towards medications with similar efficacy profile (horizonal switch) [33], as also suggested in clinical studies [24, 40].

Interferon Beta formulations have high adherence rates (around 85%), which is in line with [41, 42], or even higher than previous population-based studies [38, 43, 44], and, thus, reassuring in terms of quality of MS healthcare delivery in the Campania Region [18, 38]. In our cohort, adherence rates were lower for Betaferon®, as also described by Bartolomé-García and colleagues [39], and higher in Plegridy®, when compared with Rebif®. These results suggest that adherence is not necessarily related to frequency of administration (which is similar in Betaferon® and Rebif®, and lower in Plegridy®), but is rather multifactorial (e.g., injection device, support services) [41, 42]. Unfortunately, we were unable to assess whether differences in adherence were also associated with worse disease outcomes, as suggested by previous studies [41, 42].

A limited number of patients presented with hospital admissions during the study period. In particular, patients treated with Betaferon® presented with higher risk of MS-related hospital admissions, also resulting into higher costs, when compared with Rebif®. This result points towards sub-optimal disease control for Betaferon®, possibly also as a consequence of reduced adherence rates [26]. As such, higher costs for DMT prescription can result into better management of MS [45], with reduced number of MS-related complications, and subsequent medical and societal costs. Also, we found significantly lower costs for inpatient admissions in Plegridy®, compared with Rebif®, though possibly requiring longer follow-up to be better studied (EMA approval for Plegridy® was in 2014) [3].

Among limitations, we have to acknowledge the present study was conducted from 2015 to 2017, and, thus, our findings do not necessarily apply to the future treatment scenario of MS, which has become more complex with the introduction of new DMTs [3, 31–33]. Unfortunately, there are a number of clinical variables that can affect treatment persistence and adherence (e.g., disease duration, disability, MRI measures) [33, 41, 42], that cannot be retrieved from routinely-collected healthcare data; in the future, data linkage to clinical registries and validation of clinical outcomes should be considered. Also, results obtained in the Campania Region of Italy might not easily refer to other geographical areas, with different prescription patterns and healthcare organisation.

## Conclusions

Rebif® is more expensive than other Interferon Beta formulations, but is more frequently prescribed to young patients, and is associated with reduced risk of MS-related complications, with subsequently lower hospital admissions and costs. In the future, these results will need to be confirmed using data linkage between routinely collected healthcare datasets and clinical registries, in order to obtain detailed clinical measures and to adjust for potential confounding factors.

## Data Availability

Data is available upon request to Regional Healthcare Society (So.Re.Sa – www.soresa.it).

## Abbreviations

DMT: Disease modifying treatment
MS: Multiple sclerosis

## References

[1] Thompson AJ, Baranzini SE, Geurts J, Hemmer B, Ciccarelli O (2018). Multiple sclerosis. Lancet.

[2] Wallin MT, Culpepper WJ, Nichols E, Bhutta ZA, Gebrehiwot TT, Hay SI (2019). Global, regional, and national burden of multiple sclerosis 1990–2016: a systematic analysis for the global burden of disease study 2016. Lancet Neurol.

[3] De Angelis F, John N, Brownlee W (2018). Disease-modifying therapies for multiple sclerosis. BMJ..

[4] Petruzzo M, Palladino R, Nardone A, Nozzolillo A, Servillo G, Orlando V (2020). The impact of diagnostic criteria and treatments on the 20-year costs for treating relapsing-remitting multiple sclerosis. Mult Scler Relat Disord.

[5] Mendes D, Alves C, Batel-Marques F (2016). Benefit – risk of therapies for relapsing – remitting multiple sclerosis : testing the number needed to treat to benefit (NNTB), number needed to treat to harm (NNTH) and the likelihood to be helped or harmed (LHH): a systematic review and Meta- analysis. CNS Drugs.

[6] Fogarty E, Schmitz S, Tubridy N, Walsh C, Barry M (2016). Comparative efficacy of disease-modifying therapies for patients with relapsing remitting multiple sclerosis: systematic review and network meta-analysis. Mult Scler Relat Disord.

[7] Einarson T, Bereza B, Machado M (2017). Comparative effectiveness of interferons in relapsing-remitting multiple sclerosis: a meta-analysis of real-world studies. Curr Med Res Opin.

[8] Mitsikostas DD, Goodin DS (2017). Comparing the efficacy of disease-modifying therapies in multiple sclerosis. Mult Scler Relat Disord.

[9] Claflin SB, Tan B, Taylor BV (2019). The long-term effects of disease modifying therapies on disability in people living with multiple sclerosis: a systematic review and meta-analysis. Mult Scler Relat Disord.

[10] Moccia M, Palladino R, Carotenuto A, Saccà F, Russo CV, Lanzillo R (2018). A 8-year retrospective cohort study comparing interferon-β formulations for relapsing-remitting multiple sclerosis. Mult Scler Relat Disord.

[11] Trojano M, Tintore M, Montalban X, Hillert J, Kalincik T, Iaffaldano P (2017). Treatment decisions in multiple sclerosis - insights from real-world observational studies. Nat Rev Neurol.

[12] Kalincik T, Butzkueven H (2016). Observational data: understanding the real MS world. Mult Scler.

[13] Glaser A, Stahmann A, Meissner T, Flachenecker P, Horáková D, Zaratin P (2019). Multiple sclerosis registries in Europe - an updated mapping survey. Mult Scler Relat Disord.

[14] Torres EM, Fernández ÓF, Rueda PC, Ruiz-beato E, Pérez EE, Estrada RM (2020). Social value of a set of proposals for the ideal approach of multiple sclerosis within the Spanish National Health System : a social return on investment study. BMC Health Serv Res.

[15] Moccia M, Brescia Morra V, Lanzillo R, Loperto I, Giordana R, Fumo M (2020). Multiple sclerosis in the Campania region (South Italy): algorithm validation and 2015-2017 prevalence. Int J Environ Res Public Health.

[16] Agenzia Italiana del Farmaco (AIFA). Banca Dati Farmaci. 2020. https://www.farmaci.agenziafarmaco.gov.it/. Accessed 8 May 2019.

[17] European Medicine Agency (EMA). European public assessment reports. 2020. https://www.ema.europa.eu/en/medicines. Accessed 8 May 2019.

[18] Moccia M, Tajani A, Acampora R, Signoriello E, Corbisiero G, Vercellone A (2019). Healthcare resource utilization and costs for multiple sclerosis management in the Campania region of Italy: comparison between Centre-based and local service healthcare delivery. PLoS One.

[19] Chou IJ, Kuo CF, Tanasescu R, Tench CR, Tiley CG, Constantinescu CS (2020). Comorbidity in multiple sclerosis: its temporal relationships with disease onset and dose effect on mortality. Eur J Neurol.

[20] Charlson M, Pompei P, Ales K, MacKenzie C (1987). A new method of classifying prognostic comorbidity in longitudinal studies: development and validation. J Chronic Dis.

[21] Evans C, Tam J, Kingwell E, Oger J, Tremlett H (2012). Long-term persistence with the Immunomodulatory drugs for multiple sclerosis: a retrospective database study. Clin Ther.

[22] Zhornitsky S, Greenfield J, Koch MW, Patten SB, Harris C, Wall W (2015). Long-term persistence with injectable therapy in relapsing-remitting multiple sclerosis: an 18-year observational cohort study. PLoS One.

[23] Moccia M, Palladino R, Carotenuto A, Russo CCVCV, Triassi M, Lanzillo R (2016). Predictors of long-term interferon discontinuation in newly diagnosed relapsing multiple sclerosis. Mult Scler Relat Disord.

[24] Lanzillo R, Prosperini L, Gasperini C, Moccia M, Fantozzi R, Tortorella C (2018). A multicentRE observational analysiS of PErsistenCe to treatment in the new multiple sclerosis era: the RESPECT study. J Neurol.

[25] Lam WY, Fresco P (2015). Medication adherence measures: an overview. Biomed Res Int.

[26] Evans C, Marrie RA, Zhu F, Leung S, Lu X, Kingwell E (2017). Adherence to disease-modifying therapies for multiple sclerosis and subsequent hospitalizations. Pharmacoepidemiol Drug Saf.

[27] Soggetto Aggregatore della Regione Campania (So.Re.Sa.). Anagrafica Farmaci. 2017. https://www.soresa.it/pa/Pagine/Anagrafe.aspx. Accessed 20 Apr 2020.

[28] Battaglia M, Kobelt G, Ponzio M, Berg J, Capsa D, Dalén J (2017). New insights into the burden and costs of multiple sclerosis in Europe: results for Italy. Mult Scler.

[29] Kobelt G, Thompson A, Berg J, Gannedahl M, Eriksson J (2017). New insights into the burden and costs of multiple sclerosis in Europe. Mult Scler.

[30] San-Juan-Rodriguez A, Good CB, Heyman RA, Parekh N, Shrank WH, Hernandez I (2019). Trends in prices, market share, and spending on self-administered disease-modifying therapies for multiple sclerosis in Medicare part D. JAMA Neurol.

[31] Marangi A, Farina G, Vicenzi V, Forlivesi S, Calabria F, Marchioretto F (2020). Changing therapeutic strategies and persistence to disease-modifying treatments in a population of multiple sclerosis patients from Veneto region, Italy. Mult Scler Relat Disord.

[32] Ogino M, Okamoto S, Ohta H, Sakamoto M, Nakamura Y, Iwasaki K (2017). Prevalence, treatments and medical cost of multiple sclerosis in Japan based on analysis of a health insurance claims database. Clin Exp Neuroimmunol.

[33] Saccà F, Lanzillo R, Signori A, Maniscalco GT, Signoriello E, Lo Fermo S (2019). Determinants of therapy switch in multiple sclerosis treatment-naïve patients: a real-life study. Mult Scler.

[34] McKay KA, Evans C, Fisk JD, Patten SB, Fiest K, Marrie RA (2017). Disease-modifying therapies and adherence in multiple sclerosis: comparing patient self-report with pharmacy records. Neuroepidemiology.

[35] Melendez-Torres GJ, Auguste P, Armoiry X, Maheswaran H, Court R, Madan J, et al. Clinical effectiveness and cost-effectiveness of beta-interferon and glatiramer acetate for treating multiple sclerosis: Systematic review and economic evaluation. Health Technol Assess (Rockv). 2017;21:1–352. 10.3310/hta21520 PMC5623930 28914229

[36] Moccia M, Palladino R, Lanzillo R, Triassi M, Brescia MV (2017). Predictors of the 10-year direct costs for treating multiple sclerosis. Acta Neurol Scand.

[37] Furneri G, Santoni L, Ricella C, Prosperini L (2019). Cost-effectiveness analysis of escalating to natalizumab or switching among immunomodulators in relapsing-remitting multiple sclerosis in Italy. BMC Health Serv Res.

[38] Hansen K, Schüssel K, Kieble M, Werning J, Schulz M, Friis R (2015). Adherence to disease modifying drugs among patients with multiple sclerosis in Germany: a retrospective cohort study. PLoS One.

[39] Bartolomé-García E, Usarralde-Pérez Á, Sanmartín-Fenollera P, Pérez-Encinas M (2019). Persistence and adherence to interferon and glatiramer acetate in patients with multiple sclerosis. Eur J Hosp Pharm.

[40] Hamidi V, Couto E, Ringerike T, Klemp M (2018). A multiple treatment comparison of eleven disease-modifying drugs used for multiple sclerosis. J Clin Med Res.

[41] Moccia M, Palladino R, Russo C, Massarelli M, Nardone A, Triassi M (2015). How many injections did you miss last month? A simple question to predict interferon β-1a adherence in multiple sclerosis. Expert Opin Drug Deliv.

[42] Cohen BA, Coyle PK, Leist T, Oleen-Burkey MA, Schwartz M, Zwibel H (2015). Therapy optimization in multiple sclerosis: a cohort study of therapy adherence and risk of relapse. Mult Scler Relat Disord..

[43] Bergvall N, Petrilla AA, Karkare SU, Lahoz R, Agashivala N, Pradhan A (2014). Persistence with and adherence to fingolimod compared with other disease-modifying therapies for the treatment of multiple sclerosis: a retrospective US claims database analysis. J Med Econ.

[44] Fernández O, Agüera J, Izquierdo G, Millán-Pascual J, Ramió i Torrentà L, Oliva P (2012). Adherence to interferon β-1b treatment in patients with multiple sclerosis in Spain. PLoS One.

[45] Moccia M, Palladino R, Lanzillo R, Carotenuto A, Russo CV, Triassi M (2017). Healthcare costs for treating relapsing multiple sclerosis and the risk of progression: a retrospective Italian cohort study from 2001 to 2015. PLoS One.

